# Novel Technique for Open Surgical Tracheostomy in Small Children

**DOI:** 10.5811/westjem.2021.11.53296

**Published:** 2022-02-23

**Authors:** Nicholas S. Simpson, Kelsey M. Spaur, Ashley M. Strobel, Evan J. Kirschner, Brian E. Driver, Robert F. Reardon

**Affiliations:** * Hennepin County Medical Center, Department of Emergency Medicine, Minneapolis, Minnesota; † Elson S. Floyd College of Medicine, Department of Emergency Medicine, Spokane, Washington

Performing an emergency surgical airway on a young child is a harrowing event. Modern supraglottic devices have made the need for pediatric surgical airways exceedingly rare. Because of the rarity of pediatric surgical airways, many physicians have low confidence with performing this procedure.^1^,^2^ The best method of performing a surgical airway in children remains unclear. The pediatric cricothyroid membrane is too small to perform a traditional cricothyrotomy; in neonates, it is five times shorter and four times narrower than that of an adult, with a mean length of 2.6 +/− 0.7 millimeters (mm) and width of 3 +/− 0.63 mm.^3^,^4^ For this reason, cricothyrotomy is not recommended for children younger than about 8–10 years old.

Both transtracheal needle ventilation (TTNV) and open surgical tracheostomy are described as alternative options.^5^ Transtracheal needle ventilation (also known as transtracheal jet ventilation) has been traditionally recommended as the initial approach in the cannot intubate, cannot oxygenate (CICO) scenario; however, this recommendation has been challenged recently because there is scant evidence to support the use of TTNV in this setting. The rationale for TTNV is that it is quicker than an open surgical technique and has less risk of damage to surrounding structures. Some literature also recommends TTNV as a bridge until otolaryngologists or other experienced clinicians arrive, but many emergency physicians do not practice in environments with these resources and need to have options for definitive management. Additionally, TTNV use in emergency situations is incredibly rare, and knowledge of actual clinical performance is limited to adult patients.

The originally described options include use of a large-bore needle with a 3.0-mm inner diameter (ID) endotracheal tube adapter connected to a bag valve device, or use of a large-bore needle with a 3-milliliter syringe attached and an 8.0-mm ID endotracheal tube adapter in the barrel of the syringe. Since then, several commercially available devices were introduced; however, few are small enough for a neonate or infant cricothyroid membrane, and few have been studied in children at all.^3^ Smith et al reported two cases in which a TTNV needle and catheter were placed through an old tracheostomy fistula and scar, which is much different from using it on a child without a prior tracheostomy.^6^ Depierraz et al described TTNV use in 16 children for 28 elective cases in patients with known subglottic stenosis. They carefully placed transtracheal needles and catheters using direct endoscopic guidance and concluded that the procedure is only safe when performed in this manner. Their experience included a six-week-old child weighing only 2.8 kilograms who received TTNV using an 18-gauge, 0.8-mm inner diameter, 37-mm length Teflon catheter, and who suffered barotrauma with bilateral pneumothoraces and subcutaneous emphysema requiring tracheal intubation and bilateral chest tube placement.^7^

Barotrauma, subcutaneous emphysema hampering subsequent surgical attempts, and device failure are well described complications of TTNV. The rate of significant complications is about 50% when TTNV is attempted in CICO scenarios, and it is rarely performed in these emergent situations.^7^,^8^ A review of TTNV in the literature identified only two instances of the procedure being performed emergently in children aged 1–8 years old.^8^ In addition to the lack of evidence for its utility, TTNV requires specialized equipment that may not be available in an emergency department (ED) setting. Also, there is experimental evidence, which correlates with our laboratory and clinical experience, showing that the success rate of TTNV in small children is significantly lower than surgical tracheostomy.^9^

A review by Coté et al advocates for surgical tracheostomy.^3^,^10^ The recent movement away from TTNV should prompt those who perform emergency airway management to consider their own skill level and ability to perform an open surgical procedure and to review the details of the procedure. The emergency open pediatric surgical tracheostomy is often taught to be similar to an elective open tracheostomy, with careful dissection of soft tissues to visualize the trachea before placing stay sutures, incising the trachea, and inserting a tracheostomy tube.^11^ A review by Koers et al demonstrates the high success rate of scalpel tracheostomy, with longer procedure time being the limiting factor.^12^

Therefore, we developed a technique that was meant to be quick and successful and that we believe is the fastest way of performing an open surgical tracheostomy. This technique uses equipment that is readily available in all EDs and consists of just a few steps to a definitive airway. While teaching emergency surgical techniques in our live animal lab over the last few decades we found that the most difficult part of performing an open tracheostomy on subjects with a very small trachea (live rabbit model) was stabilizing the trachea during incision and blunt dissection of the overlying soft tissues, which is even more important in infants whose cartilage is more pliable. Therefore, we developed this technique to overcome those difficulties.

In this novel method, first, a midline vertical incision is made through the skin overlying the trachea. After using palpation to confirm tracheal location, a single 0-silk stay suture is placed in the sagittal plane in the midline through the overlying soft tissue and the trachea. Both sides of the suture are then pulled upward and held firmly by an assistant while the primary operator dissects the overlying soft tissue and exposes the trachea. Then, a horizontal incision is made in the trachea superior to the stay suture (see the Figure for anatomic details); then a bougie is inserted into the trachea, followed by an endotracheal tube.

After obtaining exemption from the Institutional Review Board and approval from the animal care committee, we used a live, anesthetized rabbit model with tracheal diameters ranging from 5–6 mm, simulating the airway of a human child aged 0–4 years old, to compare this novel technique to a standard open surgical technique. While this model accurately reflects the size of an infant or young child’s trachea, there may be less subcutaneous fat and bleeding than in humans. This highlights the importance of learning the novel technique as a tactile rather than visual procedure, which better prepares learners for the real-life experience. In the standard technique, a midline vertical incision is made over the trachea, followed by blunt dissection with forceps and blunt scissors until the trachea is visualized. Then two stay sutures are placed in the lateral trachea, and the trachea is incised vertically and a tracheostomy tube is placed into the trachea.

We enrolled 15 emergency medicine residents who had never performed tracheostomies on humans to randomly perform the novel or standard technique. Seven residents performed the novel technique, with success on the first attempt occurring in six of seven, with a median time of 239 seconds (s) (95% confidence interval [CI], 190–664 s). Eight residents performed the standard technique, with success on the first attempt occurring in seven of eight, with a median time of 306 s (95% CI, 230–439 s). There was an absolute difference between groups of 39 s (95% CI, −118 s to 164 s). No residents needed to switch from one technique to the other, and all rabbits were successfully cannulated after two or three attempts. In the novel technique, there were two instances where the faculty physician had to intervene in the procedure (both to help identify the trachea and place the stay suture in the correct location), and one instance of complete tracheal transection caused by the horizontal incision. Some faculty assistance is expected with critical procedures at an academic facility and highlights the necessity of practice and familiarity with the procedure when practicing independently.

This data highlights the fact that performing an open tracheostomy on a very small, live animal model is a difficult, time-consuming procedure that requires practice before attempting it in a child. Although this novel technique may be performed faster than a traditional open surgical tracheostomy it might be technically more difficult, as there was one instance of tracheal transection, which is a significant complication. Risk of this potentially life-ending complication could be reduced by using sharp scissors to vertically cut the trachea above the stay suture, rather than incising horizontally with a scalpel, a technique previously described to be successful.^9^ When a small child requires an emergency surgical airway, however, time is paramount, and in many instances there is not enough time to perform a careful dissection to visualize the trachea.

The fastest, most successful, and safest method of performing a surgical airway on a small child remains unknown, and more research on this procedure is needed. We believe that this study demonstrates the importance of stabilizing the trachea prior to dissection of the overlying soft tissue. It also demonstrates the importance of visualizing and carefully incising the trachea, which makes this procedure very different from an emergent cricothyrotomy in an adult and highlights the need to practice this specific procedure.

## Figures and Tables

**Figure:**
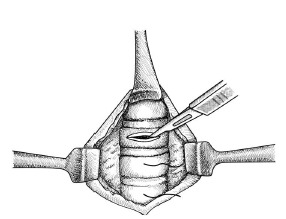
Anatomic details: The midline stay suture is placed before tracheal visualization and helps stabilize the trachea during blunt dissection. Once the trachea is seen, a horizontal incision is made superior to the stay suture. *The retractors are included for clarity of the drawing but are not necessary during the actual procedure.
